# Measuring Instruments for Media Health Literacy: A Systematic Review of Psychometric Properties

**DOI:** 10.3390/nursrep14040206

**Published:** 2024-10-06

**Authors:** Noelia Navas-Echazarreta, Raúl Juárez-Vela, Antonio Martínez-Sabater, Emmanuel Echániz-Serrano, María Teresa Fernández-Rodrigo, Olga Navarro-Martínez, Consuelo Sancho-Sánchez, Ana Cobos-Rincón, Antonio Rodríguez-Calvo, Silvia González-Fernández, Elena Chover-Sierra, Pedro José Satústegui-Dordá

**Affiliations:** 1 Doctoral Program in Health Sciences and Sports, University of Zaragoza, 50009 Zaragoza, Spain; noelia.navas@unirioja.es; 2 Department of Nursing, University of La Rioja, 26006 Logroño, Spain; 3 GRUPAC Research Group, Department of Nursing, University of La Rioja, 26006 Logroño, Spain; ana.cobos@unirioja.es; 4 Faculty of Nursing, University of Valencia, 46010 Valencia, Spain; antonio.martinez-sabater@uv.es (A.M.-S.); olga.navarro@uv.es (O.N.-M.); elena.chover@uv.es (E.C.-S.); 5 Care Research Group (INCLIVA), Hospital Clínico Universitario de Valencia, 46010 Valencia, Spain; 6 Media Literacy in Health Group (GRUPAMES)—Innovation and Training in Educational Sciences Research Center (CIFICE), University of Zaragoza, 50009 Zaragoza, Spain; maitefer@unizar.es (M.T.F.-R.); pjsd@unizar.es (P.J.S.-D.); 7 SAPIENF (B53_23R) Research Group, Department of Physiatry and Nursing, Faculty of Health Sciences, University of Zaragoza, c/Domingo Miral s/n, 50009 Zaragoza, Spain; 8 Faculty of Medicine, University of Salamanca, 37007 Salamanca, Spain; sanchoc@usal.es (C.S.-S.); sigofe@usal.es (S.G.-F.); 9 Hospital Universitario de Salamanca, 37007 Salamanca, Spain; arodriguezc@saludcastillayleon.es; 10 Internal Medicine Department, Hospital General Universitario, 46014 Valencia, Spain

**Keywords:** media health literacy, health, systematic review, scale, instrument

## Abstract

Background**:** Informational overload hinders the recognition of quality information and influences a population’s health-related decisions. In this context, media health literacy aims to promote citizens’ critical analysis skills, contributing to informed decision-making. This study aims to identify the instruments used to measure the level of media health literacy and their psychometric properties. Methods: A systematic review of the scientific literature was performed in 2023. The articles were extracted from the electronic databases “Pubmed”, “Web of Science”, “Dialnet”, and “Scopus”. The search languages were limited to English, Spanish, and Portuguese. Results: Twelve articles were selected for further analysis. The described measurement instruments included five original scales and seven cross-cultural adaptations of three of them. Four scales (the Sugar-Sweetened Beverages Media Literacy scale adapted to Turkish and Chinese, along with the Media Health Literacy (MeHLit) scale and its adaptation to the Chinese language) exhibited high quality in the assessment of psychometric properties. Conclusions: These instruments allow for the measurement of an individual’s level of skill when consuming specific health information, enabling an analysis to understand the risk they are exposed to. Further research is recommended to strengthen the existing evidence and apply these tools to broader and more diverse populations.

## 1. Introduction

Citizens remain exposed to a constant flow of information through various media channels. This prevailing information overload makes it difficult to recognize quality information or the multiple informational disorders it may include. In this regard, during the COVID-19 pandemic, the vast amount of shared information facilitated the spread of disinformation through various media outlets [1,2,3,4].

Digital media provide an ideal environment for the circulation of misinformation. The digital format facilitates the easy viralization of health topics [5,6]. Additionally, communicators and other social media users benefit from the dissemination of controversial information [7].

In this context, media literacy emerges as one of the most powerful means in the fight against misinformation, urging citizens to take responsibility [8]. Media literacy encompasses processes that enable the development of skills, abilities, and capacities to critically analyze various media, their informational content, and the social effects they provoke [9]. Empowering individuals to discern informational disorders helps them make informed decisions, especially in the field of health [8,10].

As early as 2001, Kickbusch [11] highlighted the significance of education in health promotion and disease prevention, identifying health literacy as a powerful tool to address this challenge. She also emphasized the role of mass media and electronic texts as key components of health literacy, given their function as sources of health information, and encouraged individuals to use critical thinking when consuming such content.

It was not until a decade later that Levin-Zamir et al. [12] addressed the need for the concept of health literacy to evolve to include media literacy, particularly due to the increasing prevalence of digital information. The authors argued that the ability to access, understand, and evaluate health information in the media is crucial for making informed and safe health decisions, thereby becoming an essential component of health literacy in the digital age. This perspective laid the foundation for the development of the concept of media health literacy, recognizing the interdependence between critical information management and health literacy.

In their 2011 study, Levin-Zamir et al. [12] established that media health literacy could be measured based on four dimensions, namely the identification of health-related content within messages, the influence on individuals’ health behaviors, the critical analysis of information, and the impact of media information on individuals’ health-related behaviors.

A year later, Ferrés and Piscitelli [13] proposed the dimensions and indicators that would define media competence. The rise of the digital sphere and native digital media highlighted the need to promote media education among the public, emphasizing the critical analysis of consumed media content. Their proposal is based on six dimensions, namely languages, technology, interaction processes, production and dissemination processes, ideology and values, and finally, the esthetic dimension. Each of these dimensions assessed an individual’s analytical and comprehension abilities, underscoring the importance of critical thinking and media education when consuming information from media sources.

Organizations such as the United Nations Educational, Scientific and Cultural Organization (UNESCO) and the European Union have prominently promoted media literacy since 2014 as an essential skill for contemporary society. The widespread and unlimited access to the internet, as well as digital platforms and media, necessitates a society with specific knowledge to take an active stance towards them [14].

### Differences between Health Literacy, eHealth Literacy, and Media Health Literacy

The dissemination of health misinformation has negative repercussions on individuals’ lives. The presence of such information in the media sphere renders citizens vulnerable, influencing their habits and self-care practices. Various researchers such as García-Marín [15], Guallar et al. [16], García-Saisó [1], León et al. [17], Sánchez Duarte and Magallón-Rosa [18], and the World Health Organization [19] support these claims. Digital media, particularly social networks, serve as unlimited storage spaces for information. Health is one of the primary concerns of the population and generates the highest number of searches online [20]. According to Levin-Zamir and Bertschi [10], the conception of individuals as passive subjects conditioned by various factors affecting their health, such as the media, is erroneous and diminishes the possibility of improving their self-care.

Furthermore, since health is one of the topics that generates the most interest among citizens, it becomes a highly mediated aspect with a higher likelihood of presenting informational errors that the population must be able to analyze and discern. Therefore, it is necessary to understand the main types of literacy that differ from media literacy [20].

On the one hand, health literacy, as defined by the World Health Organization [19], is described as “the cognitive and social skills which determine the motivation and ability of individuals to gain access to, understand, and use information in ways which promote and maintain good health”. Health literacy promotes the acquisition of skills to enable individuals to understand and use health information to make appropriate health decisions [6].

On the other hand, digital health literacy or eHealth literacy enables individuals to search, analyze, and understand health information obtained through electronic sources to make informed health decisions [20]. Both concepts refer to an individual’s ability to interact with health information, but they differ in the context and mediums through which this information is acquired and utilized. eHealth Literacy is a more specific type of health literacy that has emerged with the expansion of digital information and access to information and communication technologies (ICTs) [21].

Both are distinguished from media health literacy, as the latter is a synthesis of health literacy and media literacy. This literacy focuses on health information transmitted through traditional media, digital media, and all platforms or multimedia content disseminated by healthcare systems [10,22]. While health literacy and eHealth literacy focus on the understanding and use of health information, media health literacy additionally integrates the ability to critically analyze health information disseminated through mass and digital media. Media health literacy is particularly relevant in the current disinformation context, where information overload and misinformation have become structurally embedded in society. In this sense, equipping the public with this competency allows them to discern reliable media information that aids in making appropriate health decisions [10,23].

The content disseminated by these media outlets can positively or negatively impact health, underscoring the importance of media health literacy. This literacy aims to foster individuals’ critical thinking skills so they can identify health-related content transmitted by the media, analyze it, and recognize its impact on health and self-care. Understanding the extent to which the population possesses the necessary abilities and skills for analyzing media information contributes to guiding educational strategies in the fight against disinformation, particularly in the health domain [24].

In this sense, literacy programs represent an appropriate learning strategy that intervenes in media education and health education for citizens. Additionally, it is necessary to assess and measure the level of media health literacy present in each population [10,19]. To achieve this, the use of scales and measurement instruments is essential in obtaining a snapshot of the current context.

Media health literacy constitutes a measurable concept through various characteristics or indicators that identify it [20]. Media health literacy is becoming increasingly relevant in the digital age, where individuals are exposed to a vast amount of media information containing health-related messages. However, despite the growing interest in this area, there is a lack of consensus on how to effectively measure media health literacy, which complicates the comparison of studies and the application of results in practice.

Therefore, the research question that motivated the present systematic review was as follows: what measurement instruments exist to assess media health literacy in individuals aged 12 to 65 years, and what are their psychometric properties? Although this research question may seem ambitious, it is essential to guide the investigation towards a thorough analysis of the available instruments that, along with their psychometric properties, offer a value that determines the level of media health literacy. This approach narrows the study to these instruments, excluding tools that assess media health literacy through other methods and in other age groups. This method not only helps identify what instruments are available for a broad population range but also provides a better understanding of the characteristics and applicability of these instruments in diverse contexts, which is crucial for advancing research and practice in media health literacy.

This review was designed to address this gap, providing a critical and comprehensive review of the psychometric instruments used in previous studies. It not only maps the current landscape of available tools but also aims to identify weaknesses and strengths in the existing measures, which can guide future research and the improvement of current tools. This study presents a current overview that is essential for moving towards a more standardized and robust approach to measuring the level of media health literacy in the population.

Taking into account the aforementioned concerns, this study aims to identify the instruments used to measure the level of media health literacy in the population older than 12 years and their psychometric properties.

## 2. Materials and Methods

### 2.1. Study Design

A systematic review of the scientific literature was conducted, defined as an integrative, observational, retrospective, and secondary study that combines studies related to a specific research question [25]. The systematic review is a rigorous method for synthesizing existing evidence in a field of study. In this regard, Denyer and Tranfield [26] argue that this approach is particularly valuable in areas where the literature is broad and diverse, as in the present topic of study. The systematic review allows for the structured identification, evaluation, and synthesis of research [26]. This approach not only ensures reproducibility and transparency in the review process but also helps to map the available media health literacy measurement instruments and identify gaps in existing knowledge.

In this regard, it was decided to follow the guidelines provided by the latest update of the PRISMA statement in 2020 [27] (Supplementary Material Table S1). Before commencing the study, the research protocol for the systematic review was registered in the Prospective International Registry of Systematic Reviews (PROSPERO) with registration number CRD42023488159.

The current systematic review aims to gather, summarize, and analyze the various measurement scales of media health literacy present in the scientific literature, utilizing the Consensus-based Standards for the Selection of Health Status Measurement Instruments (COSMIN) for systematic reviews [28].

### 2.2. Search Strategy

A search was performed in the following electronic databases: “Pubmed”, “Web Of Science”, “Dialnet”, and “Scopus.” The free and “Mesh” terms used were “media health literacy”, “media literacy”, “health literacy”, “communications media”, “information literacy”, “health”, “health literacy”, “questionnaire”, “scale”, “measurement”, “instrument” or “tool” or “scale” or “questionnaire”. The search strategy was conducted by combining the OR and AND operators. The documents collected were limited to English, Spanish, and Portuguese. These databases were searched from their inception until 1 January 2024.

The selection of databases was based on their wide recognition and acceptance in the academic community and their comprehensive coverage of the relevant literature in the field of media health literacy. Additionally, the selection of search terms was conducted following a preliminary literature review to ensure the inclusion of the most relevant studies.

Through the collected articles, a reverse search was also carried out for the collection of articles of interest. These were grouped according to the type of study and study variables (most commonly used tools; media health literacy) to establish and evaluate the evidence.

“Mendeley” (Version 1.19.8) bibliographic management software was used to handle the documents retrieved in the search. The search strategy used to select the documents comprising this systematic review, as well as the terms employed, the search period, and the articles obtained, are displayed in Table 1.

### 2.3. Selection Criteria

The studies included in the present systematic review were those that addressed the development, validation, and/or use of the psychometrics of a media health literacy measurement instrument directly or indirectly. The population assessed comprised individuals aged over 12 years and under 65 years. Types of studies included systematic reviews, observational studies, and cross-sectional studies.

Furthermore, we excluded studies that did not measure media literacy in health, such as research that only studied other types of related literacy (eHealth literacy, health literacy, or media literacy alone). The following types of publications were also excluded: editorials, letters, legal cases, interviews, book chapters, commentary articles, news, review studies, and methodological considerations. Research not conducted on humans, duplicate studies, and studies in languages other than English, Spanish, and Portuguese were also excluded.

### 2.4. Effect Measures

A methodological quality assessment was conducted in three phases. Firstly, the quality of each study was evaluated based on its design using the STROBE scale (“Strengthening the reporting of observational studies in epidemiology”) [29].

In the second stage, the bias level was assessed using the COSMIN Risk of Bias checklist [28]. This tool enabled the classification of the quality of each study into a ranking of four scores ranging from “very good”, “adequate”, and “doubtful” to “inadequate”. The final quality score for each study is assigned by selecting the lowest obtained score.

Afterward, to analyze the psychometric properties evaluated in each study, the COSMIN checklist (Consensus-based Standards for the Selection of Health Status Measurement Instruments) [28] for psychometric properties of health status measurement questionnaires was utilized. The COSMIN guideline is based on the criteria for good measurement properties by Terwee et al. [30]. Thus, the psychometric properties of the different scales were scored as sufficient (+), insufficient (−), or indeterminate (?).

Once the properties evaluated in each research have been indicated, a synthesis of the strength of evidence possessed by each study regarding the evaluation of the psychometric properties will be conducted. This will be classified into the following levels: “high”—strong—(excellent methodological quality study), “moderate” (good methodological quality study), “low”—limited—(adequate methodological quality study), or “very low”—unknown—(poor methodological quality study).

### 2.5. Data Extraction (Selection and Codification)

The initial selection of documents was carried out systematically, starting with a title evaluation to determine superficial relevance, followed by a detailed reading of abstracts to ensure that the studies met the pre-established inclusion criteria. A first investigator extracted the data into an Excel spreadsheet, as recommended by the COSMIN checklist [31], which is widely recognized in the evaluation of psychometric properties, ensuring consistency and quality in the collection of relevant data. This process was subsequently verified by a second author. A third investigator, with experience in systematic reviews and psychometric property evaluation, acted as a reviewer in case of discrepancies between both investigators making the final decision after thorough analysis. The data collected from each of the studies were standardized into predefined categories, which included the country, study design, objective, population, measurement instrument, instrument properties, tests, and statistical results obtained, along with the study conclusions. This process facilitated the comparison of the studies and their subsequent analysis.

### 2.6. Data Summarization Strategy

A narrative synthesis of the results of the included studies was conducted, structured according to the type of measurement instrument and its psychometric properties.

## 3. Results

**Figure 1:**
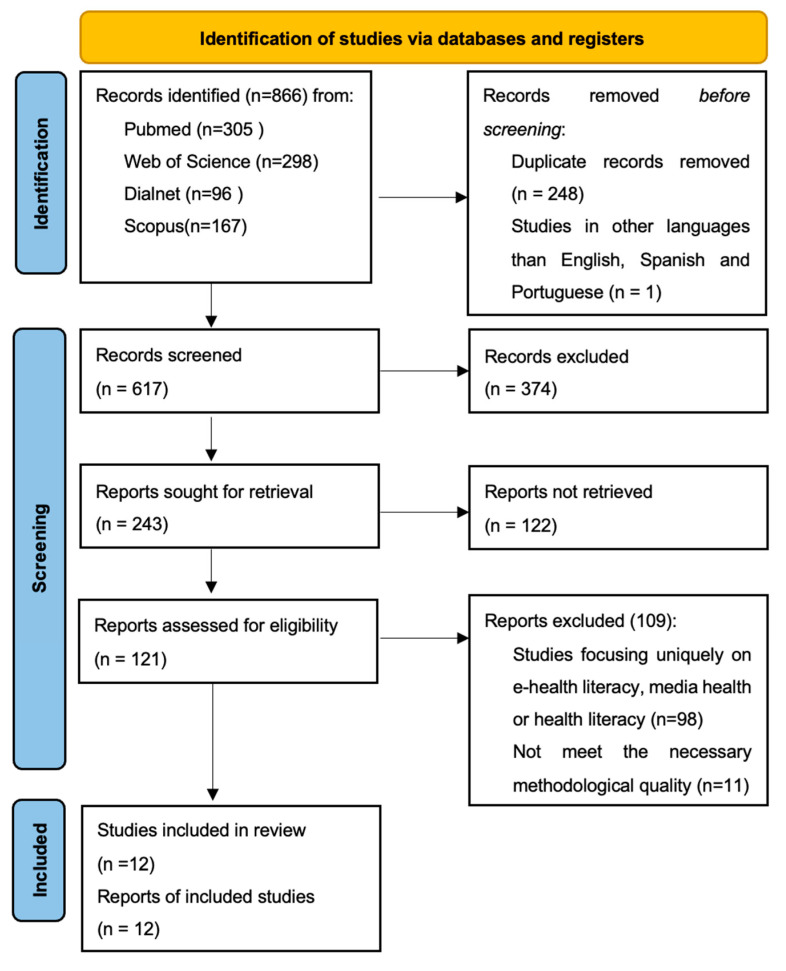
Selection process flow diagram.

The initial search yielded a total of 866 articles, of which 12 were finally selected for the systematic review (Figure 1).

As for the study design, all were characterized by a descriptive cross-sectional design. According to the country where they were conducted, three were carried out in the United States [32,33,34], one in Vietnam [35], one in Hungría [36], one in Israel [12], one in Turkey [37], one in Korea [38], two in China [39,40] and two in Iran [41,42].

All studies included population samples larger than 200 subjects. Out of the twelve studies, five assessed the properties of original scales [12,32,33,41,42]. The remaining seven conducted a cross-cultural adaptation and validation in different populations.

A summary of the articles selected for this systematic review can be found in the table in Appendix A.

### 3.1. Evaluation of the Level of Bias

The level of bias was analyzed using the COSMIN scale, which measured the quality of the studies, their design, and the use of testing procedures in scale construction. As shown in Table 2, the results of the bias level analysis demonstrate mostly doubtful quality outcomes. The studies by Nazarnia et al. [41] and Li et al. [40] were the only ones rated as having adequate quality in their final score. Conducting a pilot test was deemed inadequate in the studies by Chen et al. [33] and Jormand et al. [42]. On the other hand, in three out of the twelve studies [32,35,36], pilot testing was deemed doubtful, and no data on pilot testing were collected in the study by Demir et al. [37].

### 3.2. Measuring Instruments

The systematic literature search yielded twelve studies on measurement scales for media health literacy. Among them, as previously mentioned, the Smoking Media Literacy Scale for Adolescents (SML) by Primack et al. [32] was translated, adapted, and validated into Vietnamese [35], Hungarian [36], and Korean [38]. The latter, the Korean version of the Smoking Media Literacy Scale for Adolescents (K-SMLS), was adapted and validated in adolescent populations. Subsequently, Levin-Zamir et al. [12] validated the Media Health Literacy (MHL) scale in the Jewish adolescent population in Israel, which was adapted and validated for clinical and research settings in the adolescent population in the United States in the study by Fleary [34]. The Sugar-Sweetened Beverages Media Literacy (SSB-ML) scale by Chen et al. [33]. was translated, adapted, and validated in two studies in different countries, targeting the Turkish population [37] and the Chinese population [39]. Similarly, the Media Health Literacy (MeHLit) scale by Nazarnia et al. [41] was validated in the Chinese language a year later [40].

The COVID-19 Media Literacy scale (C-19ML) was developed by the authors [42] based on a review of the existing scientific literature, similar to authors Primack et al. [32], Levin-Zamir et al. [12], Chen et al. [33], and Nazarnia et al. [41], for their respective original scales.

Regarding the item content, they were grouped into the same dimensions (Authors and Audiences, Messages and Meanings, Representation and Reality) both in the SSB-ML scale [33] and its subsequent transcultural adaptations, as well as in the K-SMLS scale [38]. However, the latter differs from its original scale in terms of dimensions, as shown in Table 3.

The Sugar-Sweetened Beverages Media Literacy instrument, both the original version [33] and its transcultural adaptations [37,38], utilized a seven-point Likert-type response scale ranging from strongly disagree (1) to strongly agree (7). Three scales were based on a five-point Likert scale as follows: MeHLit, ranging from never (0) to always (4) [41] and its adaptation to the Chinese language [40] and the COVID-19 media literacy scale by Jormand et al. [42], which ranged from completely disagree (1) to completely agree (5). Additionally, the SML scale [32] and its adaptations to Vietnamese and Hungarian were based on a four-point Likert-type scale with items ranging from strongly disagree to strongly agree, and the Korean version [38] ranged from strongly disagree (0) to strongly agree (3). Finally, the Adolescent Media Health Literacy scales (Adolescent MHL) by Fleary [34] employed images as items. These images conveyed a health message, and each was associated with a question with different response options depending on the encompassed dimension.

The characteristics of the measurement tools analyzed previously and their psychometric properties are synthesized in Table 3.

**Table 3:** Psychometric properties of the scales for measuring media health literacy.

Cite	Scale	Language	Target Population	Previous Scale	Number of Dimensions and Items	Dimensions	Type of Scale and Response	Results of Psychometric Properties
Primack et al. [32]	Smoking Media Literacy Scale for Adolescents (SML)	English	1211 high school students (14 to 18 years)	Own elaboration based on the available literature on media literacy on the one hand, and tobacco consumption on the other.	1-factor scale with 18 items	1-factor scale	The 4-point Likert-type scale (strongly disagree, disagree, agree, strongly agree). The scale is 10 points by dividing the raw score of 54 points by 5.4.	Internal consistency: -Cronbach’s α ^1^ = 0.87
Page, et al. [36]	Smoking Media Literacy in Vietnamese Adolescents	Vietnamese	2000 students in grades 10–12 in two high schools (15–19 years old)	Smoking Media Literacy Scale [32]. Cross-cultural adaptation.	1-factor scale with 18 items	1-factor scale	The 4-point Likert-type scale (strongly disagree, disagree, agree, strongly agree). The scale is 10 points by dividing the raw score of 54 points by 5.4.	Internal consistency: -Cronbach’s α = 0.75
Page et al. [37]	Media literacy and cigarette smoking in Hungarian adolescents	Hungarian	546 students (13–18 years old)	Smoking media literacy [32] cross-cultural adaptation	1-factor scale with 18 items	1-factor scale	The 4-point Likert-type scale (strongly disagree, disagree, agree, strongly agree). The scale is 10 points, dividing the raw score of 54 points by 5.4.	Internal consistency: -Cronbach’s α = 0.78
Levin-Zamir et al. [12]	Media Health Literacy (MHL)	English	Jewish adolescents	Own elaboration	4 dimensions, 6 item	1. Content Identification 2. Perceived influence on behavior 3. Critical analysis 4. Intended action/reaction	Items were measured on a 5-point Likert scale from 0 (no identification) to 4 (action/interaction mentioned). The final score was composed of the sum of the item results (0–24 points).	Internal consistency: -Cronbach’s α = 0.74 Reliability: the coefficient of reproducibility was 0.84 Scalability: -coefficients of scalability ranged from 0.54 to 0.80
Chen, et al. [33]	Sugar-Sweetened Beverages Media Literacy scale (SSB-ML).	English	Adultos (>18 años) consuming > 200 SSB kcal/day	Smoking Media Literacy Scale [32]	3 dimensions, 18 items	1. Authors and Audiences 2. Messages and Meanings 3. Representation and Reality	The 7-point Likert-type scale ranging from strongly disagree (1) to strongly agree (7).	Content validity: -Two rounds of revision Internal consistency: -Cronbach α = 0.89
Demir, et al. [37].	Turkish Sugar-Sweetened Beverages Media Literacy scale (Turkish SSB-ML).	Turkish	Adults (university students)	Sugar-Sweetenes Beverages Media Literacy scale (SSB-ML); Chen et al. [33] Cross-cultural adaptation	3 sub-dimensions y 19 items	1. Authors and Audiences 2. Messages and Meanings 3. Representation and Reality	The 7-point Likert-type scale. Each item is scored as “1 = absolutely disagree” “4 = neutral”, and”7 = strongly agree”.	Content validity: -CVI ^2^ = 0.96 Construct validity: -KMO ^3^ = 0.834 -RMSEA ^4^ was <0.08 CFI ^5^ = 0.94 TLI ^6^ = 0.9 4 Internal consistency: -Cronbach’s α = 0.86. Reliability: -Spearman–Brown coefficient = 0.73
Kim et al. [38]	Korean Version of the Smoking Media Literacy Scale for Adolescents (K-SMLS).	Korean	Adolescents	Smoking Media Literacy Scale [32] Cross-cultural adaptation	3 dimensions, 15 items	1. Authors and audiences 2. Messages and meanings 3. Representation and reality	4-point Likert-type scale (0 = strongly disagree, 1 = disagree, 2 = agree, and 3 = strongly agree). Total raw scores range from 0 to 54. The total scores were converted to a 10-point scale by dividing the raw score for the 54-point scale by 5.4.	Content validity: -CVI = 0.78 Construct validity: -KMO = 0.79 -CFI = 0.93 -TLI = 0.92 -RMSEA = 0.09 -SRMR ^7^ = 0.09 Internal consistency: -Cronbach’s α = 0.78 -McDonald’s Omega = 0.78
Long and Yoon [39].	Chinese Sugar-Sweetenes Beverages Media Literacy scale (C-SSB-ML).	Chinese	Adults (university students)	Sugar-Sweetened Beverages Media Literacy scale (SSB-ML) Chen et al. [33]. Cross-cultural adaptation	3 sub-dimensions, 19 items	1. Authors and Audiences 2. Messages and Meanings 3. Representation and Reality	The 7-point Likert-type scale. Each item is scored as “1 = absolutely disagree” “4 = neutral”, and ”7 = strongly agree”.	Content validity: -CVI = 0.88. Construct validity: -KMO = 0.93 -CFI = 0.92 -TLI = 0.91 -RMSEA < 0.08 -SRMR < 0.07 Internal consistency: -Cronbach’s α = 0.92 Reliability: -Spearman–Brown coefficient = 0.83 Criterion validity: -Correlation between C-SSB-ML y eHEALS (*p* < 0.001)
Fleary [34]	Adolescent Media Health Literacy scales (Adolescent MHL).	English	American adolescents	MHL [12].	3 dimensions, 21 items	1. Recognition/identification (9 items) 2. Influence/critical analysis (9 items) 3. Action/reaction (3 items)	The items are 21 images about health. -Recognition/identification (9 items): the following question is associated with each picture: “Is there a health-related message in the picture?”. Dichotomous answer: Yes/No -Influence/critical analysis (9 items): 4 response options (score 0–4). -Action/reaction (3 items): 5 response options (scored from 0–3).	Internal consistency: -KR-20 α ^8^ = 0.74 Criterion validity: -Correlation with NVS ^9^ scale (r = 0.3, *p* > 0.01) y eHEALS ^10^ (r = 0.22, *p* < 0.001).
Nazarnia, et al. [41]	Media Health Literacy (MeHLit)	English	Adults	Own elaboration based on a literature review combining keywords of media literacy and health.	5 dimensions, 21 item	1. Goal appraisal skill 2. Content appraisal skill 3. Implicit meaning appraisal skill 4. Visual comprehension skill 5. Audience appraisal skill	The 5-point Likert scale ranging from never (0), rarely (1), sometimes (2), most of the time (3), and always (4). The scoring ranges from 0 to 84 (the higher score means that a person understands more messages related to health issues).	Content validity: -CVI = 0.93 Construct validity: -KMO index was 0.896 -RMSEA = 0.051 -IFI = 0.92 -CFI = 0.93 Internal consistency: -Cronbach’s α = 0.91
Li, et al. [40]	The Chinese version of Media Health Literacy (MeHLit)	Chinese	Adults	MeHLit de Nazarnia Zarei et al. [36]. Cross-cultural adaptation	5 dimensions, 21 item	1. Goal appraisal skill 2. Content appraisal skill 3. Implicit meaning appraisal skill 4. Visual comprehension skill 5. Audience appraisal skill	The 5-point Likert scale ranging from never (0), rarely (1), sometimes (2), most of the time (3), and always (4). The scoring ranges from 0 to 84 (the higher score means that a person understands more messages related to health issues).	Content validity: -CVI = 0.85 Construct validity: -KMO = 0.77 -RAMSEA = 0.03 -SRMR < 0.07 -CFI = 0.98 -TLI = 0.97 -AVE ^11^ = 0.72 Internal consistency: -Cronbach’s α = 0.85 -McDonald’s omega = 0.83 Reliability: -Split-half = 0.9 -Test–retest = 0.9
Jormand, et al. [42].	COVID-19 Media Literacy scale (C-19ML)	English	Adults (students from a medical university)	Own elaboration based on the guide Media Literacy Training Center of the American CML [43].	5 dimensions, 21 items	1. Constructedness of credible COVID-19 media messages 2. Contractedness of fake media coronavirus messages 3. Audience 4. Format 5. Represented lifestyles in fake media coronavirus messages	The 5-point Likert scale ranging from completely disagree (1) to completely agree (5). The scoring ranges for each dimension were 4–20, 6–30, 7–35, 8–40, and 8–40. The higher scores indicated a higher C-19ML.	Content validity: -CVI = 0.94 Construct validity: -KMO = 0.86 -RAMSEA=0.093 -CFI = 0.89 -ICC ^12^ = 0.89 -AVE > 0.70 Internal consistency: -Cronbach’s α = 0.86

^1^ α = alpha; ^2^ CVI = content validity index; ^3^ KMO = Kaiser–Meyer–Olkin; ^4^ RMSEA = root mean square error of approximation; ^5^ CFI = comparative fit index; ^6^ TLI = Tucker–Lewis index; ^7^ SRMR = standardized root mean square residual; ^8^ KR-20 α = Kurder–Richardson Formula 20 (KRK-20) alpha; ^9^. NVS = Newest Vital Sign scale; ^10^ eHEALS = eHealth Literacy scale [39]; ^11^ AVE = average variance explained; ^12^ ICC = intraclass correlation coefficient.

### 3.3. Psychometric Properties of the Instruments

#### 3.3.1. Internal Consistency

Internal consistency was assessed in all studies included in the present review. Of these, 11 investigations evaluated it using Cronbach’s alpha, which was found to be greater than 0.74 in all of them, indicating satisfactory internal consistency [32,33,34,35,36,37,38,39,40,41,42]. Additionally, the Adolescent Media Health Literacy scales by Fleary [34] utilized the Kuder–Richardson Formula 20 (KR-20) alpha to measure internal consistency and obtained a result ranging from 0.74 to 0.91 for each of its dimensions, indicating very adequate internal consistency.

#### 3.3.2. Reliability

Reliability was assessed in four studies, all of which exceeded a value of 0.7 in the tests conducted. In the study by Levin-Zamir et al. [12], reliability was evaluated through the reproducibility coefficient, which obtained a value of 0.84. For the Turkish version of the SSB-ML scale [37], reliability was assessed using the Spearman–Brown coefficient, yielding a value of 0.73, similar to the Chinese version of this scale (Spearman–Brown coefficient = 0.83). The Chinese adaptation of the MeHLit scale [40] evaluated reliability through a test–retest, which showed a value of 0.9.

#### 3.3.3. Content Validity

Regarding content validity, this was measured in six studies [37,38,40,41,42]. The CVIs from these six studies obtained values higher than 0.78.

#### 3.3.4. Structural Validity

Regarding construct validity, this was measured in six studies using the Kaiser–Meyer–Olkin (KMO) statistic, which was higher than 0.77 [37], indicating that the sampling was adequate and factorial analysis could be applied to the data. Confirmatory factor analysis was included in six of the selected studies [37,38,39,40,41,42] corresponding to the SSB-ML, K-SMLS, Turkish SSB-ML, C-SSB-ML, the Chinese version of Media Health Literacy (MeHLit), and the COVID-19 Media Literacy scale (C-19MLs). For this purpose, incremental fit indices CFI (comparative fit index) and TLI (Tucker–Lewis index) were measured, with values exceeding 0.89 for CFI and exceeding 0.91 for TLI, indicating optimal fit.

Also, model fit was measured in six of the scales using the root mean squared error of approximation (RMSEA), for which values between 0.05 and 0.09 were obtained, and the standardized root mean square residual (SRMR), with values between 0.07 and 0.09, indicating a good fit [33,37,38,39,40,41,42].

#### 3.3.5. Hypothesis Testing for Construct Validity

The SML scale, both in its original version [32] and in the cross-cultural adaptations [35,36], yielded results consistent with the hypotheses regarding the direct relationship between media literacy and smoking attitudes. Likewise, the Adolescent MHL [34] and MeHLit [40,41] scales showed a positive agreement between the measurement results obtained and the hypotheses posed for media health literacy.

#### 3.3.6. Cross-Cultural Validity

This COSMIN property was evaluated in the translated versions of the scales. Regarding the Vietnamese version of the SML, cross-cultural validity was not adequate. The difference in the internal consistency reliability of the SML scale between the Vietnamese sample (Cronbach’s alpha = 0.75) and the American sample (Cronbach’s alpha = 0.87) could be attributed to cultural variations affecting item responses. Similarly, the Hungarian version of the SML showed limiting results in internal consistency (Cronbach alpha = 0.78) compared to the American study. Likewise, the Korean version [38] obtained lower internal consistency values (Cronbach’s alpha = 0.78) than the original SML. Regarding the SSB-ML [33], its Turkish version showed adequate cross-cultural validity by obtaining internal consistency values (Cronbach’s alpha = 0.86) very similar to the original scale (Cronbach’s alpha = 0.89) and satisfactory values for construct validity. Similarly, the Chinese version [39] obtained very adequate results for construct validity and exceeded the internal consistency value of the original version (Cronbach’s alpha = 0.92). Regarding the MeHLit [41], its translation into Chinese [40] showed a confirmatory factor analysis with a very adequate and stronger fit index than the original scale, guaranteeing its cross-cultural adaptation, although the internal consistency values (Cronbach’s alpha = 0.85) did not surpass those of the original scale (Cronbach’s alpha = 0.91).

#### 3.3.7. Criterion Validity

Criterion validity was evaluated in two studies for the C-SSB-ML [39] and Adolescent MHL [34] scales. In the study by Long and Yoon [39], the correlation between the C-SSB-ML scale and the eHealth Literacy Scale (eHEALS) by Norman and Skinner [44] was assessed, obtaining a satisfactory p-value with a result lower than 0.001. Similarly, Fleary [34] evaluated the correlation between her scale and the Newest Vital Sign (NVS) scales by Weiss et al. [45] and the eHEALS, for which a p-value lower than 0.001 was also obtained.

#### 3.3.8. Evaluation of Evidence

Quality was assessed using the STROBE scale, as these were cross-sectional studies. The selected articles showed high quality, as shown in Appendix A, with a STROBE score higher than 17 points.

Regarding the evaluation of the psychometric properties of the scales, as shown in Table 4, seven studies adequately assessed content validity and expressed the results quantitatively [33,37,38,39,40,41,42] and six (50%) obtained a positive assessment for structural validity [37,38,39,40,41,42]. Internal consistency was evaluated in 100% of the studies and four (33.3%) evaluated the reliability property [12,37,39,40]. Six studies (50%) obtained results for construct validity consistent with the hypotheses proposed [32,34,35,36,40,41]. Regarding cross-cultural validity, three studies showed adequate results [37,39,40], while in three others (25%), they were insufficient [35,36,38], and the rest were not estimated. Three studies (25%) adequately assessed criterion validity [12,34,39] and lastly, 100% of the reviewed studies did not assess either measurement error or responsiveness.

### 3.4. Synthesis of Quality of the Evidence Obtained

After reviewing the twelve articles included in the present systematic review, the most evaluated properties were internal consistency in 100% of the studies, structural validity (50%), hypothesis testing for construct validity (50%), and reliability (41.6%), as indicated in Table 5.

The six studies that analyzed structural validity [37,38,39,40,41,42] showed a high level of quality for this property. For internal consistency, all twelve studies exhibited high quality. Four studies [12,37,39,40] demonstrated high quality for reliability, while one showed moderate quality [41]. Six studies demonstrated high quality in hypothesis testing for construct validity [29,32,34,35,36,40,41].

Regarding cross-cultural validity, three studies evaluated it with high quality [37,39,40], while four studies showed limited or conflicting quality for this property [34,35,36,38].

Regarding criterion validity, two studies assessed it with high quality [34,39], while one study had moderate quality [12]. However, for the assessment of measurement error and responsiveness, 100% of the studies showed poor quality.

Based on these results, out of the twelve studies included in the review, four demonstrated high quality regarding the overall evaluation of the properties, with a percentage of strong evidence exceeding 50% [37,39,40,41]. These studies correspond to the Sugar-Sweetened Beverages Media Literacy (SSB-ML) scale, specifically its cross-cultural adaptations to Turkish and Chinese (C-SSB-ML), and the Media Health Literacy (MeHLit) scale and its adaptation to Chinese (Chinese-MeHLit).

**Table 5:** Summary of strength of evidence of each study.

Instrument	Article	Structural Validity	Internal Consistency	Reliability	Measurement Error	Hypothesis Testing for Construct Validity	Cross-Cultural Validity/Measurement Invariance	Criterion Validity	Responsiveness	% Strong to Moderate Evidence
SML	Primack et al. [32]	U ^1^	S ^2^	U	U	S	U	U	U	25%
SML in Vietnamese Adolescents	Page et al. [35]	U	S	U	U	S	C ^3^	U	U	25%
SML in Hungarian Adolescents	Page et al. [36].	U	S	U	U	S	C	U	U	25%
MHL	Levin-Zamir, et al. [12]	U	S	S	U	U	U	M ^5^	U	37.5%
SSB-ML	Chen et al. [33]	U	S	U	U	U	U	U	U	12.5%
Turkish SSB-ML	Demir et al. [37]	S	S	S	U	U	S	U	U	50%
K-SMLS	Kim et al. [38]	S	S	U	U	U	L ^4^	U	U	25%
C-SSB-ML	Long and Yoon [39]	S	S	S	U	U	S	S	U	62.5%
Adolescent MHL	Fleary [34]	U	S	U	U	S	L	S	U	37.5%
MeHLit	Nazarnia et al. [41]	S	S	M	U	S	U	U	U	50%
Chinese-MeHLit	Li et al. [40]	S	S	S	U	S	S	U	U	62.5%
C-19ML	Jormand et al. [42]	S	S	U	U	U	U	U	U	25%
Evidence	% strong–moderate	50%	100%	41.6%	0%	50%	25%	25%	0%	
	% limited conflicting	0%	0%	0%	0%	0%	33.3%	0%	0%	
	% unknown	50%	0%	58.4%	100%	50%	41.7%	75%	100%	

^1^ U = unknown; ^2^ S = strong; ^3^ C = conflicting; ^4^ L = limited; ^5^ M = moderate.

## 4. Discussion

The present systematic review has allowed us to synthesize and group the existing scientific evidence on the psychometric properties of media health literacy measurement scales, a concept coined relatively recently and of rigorous relevance given the vast amount of health information disseminated through the digital sphere [20,46].

In the face of the inability to correct all the informational disorders contained in the health information disseminated, media health literacy emerges as a response to assist the public in making informed decisions [10,20,47]. However, the present study highlights the limited number of measurement scales for this type of literacy. Most are reliable instruments, with their psychometric properties adequately evaluated, as demonstrated in this review following the recommendations of the COSMIN guidelines [28,30].

These scales contain simple and easy-to-understand items, which is crucial for understanding this concept that encompasses some abstract dimensions or subjective skills such as identifying the implicit meaning of the message. The scores obtained on these scales aim to indicate the level of knowledge that an individual or certain population groups have to critically analyze health information disseminated in the media [33,37,38,39,40,41,42]. Thanks to them, it is possible to analyze the risk to which these individuals are exposed and consequently, to implement actions against misinformation [10,33,37,38,40,41,42].

Knowledge about health, as well as the issues that arise in this regard, are conditioned by socioeconomic determinants and the educational background of the individual, with such knowledge being lower in the most disadvantaged population groups [48]. After the increase in access to information through the development of technology and the widespread arrival of the Internet globally, the transfer of knowledge to society has increased [20].

In 2014, the study by Zoellner et al. [49] revealed a direct positive relationship between health literacy (HL) and the ability to interpret messages disseminated in the media about sugar-sweetened beverages (SSB). Subsequently, the study by Afshar et al. [20] demonstrated the correlation between health literacy and media literacy through their dimensions, as well as the statistically significant association between the level of media literacy and factors such as gender, education received, socioeconomic status, consumption of health-focused media, or the presence of a healthcare professional in the family [44].

According to SotoudehRad et al. [46], the measurement of media literacy in health employs items based on the exploration of the author and the audience targeted by health messages, as well as the meaning of these messages and their implications in the current context (representation and reality) from a critical thinking perspective.

Thus, Nazarnia et al. [41] developed their measurement instrument (MeHLit) by grouping its items according to the dimensions of the Media Health Literacy (MHL) scale by Levin-Zamir et al. [12], similar to Fleary’s scale [34]. However, unlike MHL, the instrument of Nazarnia et al. [41] was based on the individual’s critical analysis ability, and from this premise, the dimensions were oriented according to the different aspects characterizing information, such as the message’s objective, content, implicit meaning, and target audience. On the other hand, the dimensions proposed in the SSB-ML scale were retained in its cross-cultural adaptations to Turkish (Turkish SSB-ML) by Demir et al. [37] and to Chinese (C-SSB-ML) by Long and Yoon [39], but they were also used as a reference for the adaptation to Korean [38] of the SML scale by Primack et al. [32].

Based on the evaluation of the psychometric properties conducted in the present systematic review, the object of measurement (level of media health literacy), the definition of this type of literacy, namely an individual’s skills and abilities to critically analyze information [20], and the set of dimensions and indicators that describe it, the Media Health Literacy (MeHLit) scale by Nazarnia et al. [41] exhibited high quality, which was further confirmed in its cross-cultural adaptation to Chinese [40].

These measurement instruments ensure an objective and informed decision-making process within a broad and subjective framework such as misinformation. Furthermore, based on the results, it is possible to promote the targeting and orientation of educational, communicative, and health actions to act specifically within society, considering the context and the population to which they are applied [20,50,51].

While all dimensions are related and the scores measure practically the same parameters related to the level of media literacy, not all scales have been validated in the same population group. This fact can influence the interpretation of the scores. In this regard, the Media Health Literacy (MeHLit) scale provides an advantage when applied to different population groups by broadly targeting the adult population and focusing on the individual’s critical analysis ability, describing the main characteristic of a literate subject [10,41]. Being a recently developed instrument, it has only been applied in one subsequent study by the same authors [24], which supports its suitability and effectiveness in measuring this concept. Additionally, its cross-cultural adaptation to Chinese [40] demonstrated adequate psychometric properties for measuring media health literacy and high quality in the evaluation of cross-cultural validity.

In general, these scales enable the detection of individuals with a low level of literacy and therefore are more exposed to the potential negative influence of the information they consume on their health [10,13,24].

However, there are some limitations that the authors of the present study are aware of. Firstly, the included research is of observational design and therefore may involve a higher number of biases, such as participant selection or confounding bias for uncontrolled variables. Secondly, the absence of previous systematic reviews compiling assessment or measurement scales for this specific type of literacy poses a limitation and at the same time a strength for the present systematic review. Additionally, the lack of a greater number of studies using these scales and their limited application in more heterogeneous populations pose a limitation when extrapolating the results and supporting their effectiveness.

## 5. Implications for Nursing Practice

This review assesses the psychometric characteristics of various media health literacy scales, providing a summary of the existing evidence. The results reveal a range of validated and dependable scales, which enable healthcare providers to effectively evaluate patients’ capacity to critically assess media information. Access to this information is indispensable for tailoring communication and health education to diverse population segments. The prevalence of information disorder in media content poses a public health risk by contributing to misinformation.

Adequate media health literacy empowers patients to make well-informed decisions, thereby enhancing their self-care. Furthermore, gauging the population’s media health literacy level can inform the development and implementation of targeted, customized educational initiatives. Nursing professionals, as pivotal figures in healthcare, should actively advocate for this proficiency among their patients and communities. This reinforces the role of nurses as educators and public health proponents. Instituting policies and initiatives that support health-focused media literacy contributes to high-quality care and fosters ongoing enhancement in nursing practice.

## 6. Conclusions

Of the scales compiled in the present systematic review, four demonstrated high quality in the evaluation of psychometric properties for measuring the level of media health literacy. Specifically, the MeHLit scale, the Chinese version of MeHLit, and the cross-cultural adaptations of the SSB-ML scale to Turkish and Chinese exhibited greater methodological quality in assessing their psychometric properties, as well as a higher number of properties analyzed.

This review highlights the need for further research to strengthen the existing evidence on the psychometric properties of these scales through their implementation in studies with larger and more heterogeneous population samples. The use of media health literacy measurement tools allows for an objective understanding of the population’s situation. In this way, multiple interventions can be carried out to improve knowledge and analytical skills regarding health information present in various media outlets.

## Figures and Tables

**Table 1:** The search strategy.

Database	Search String	Documents Retrieved	Documents Selected
Pubmed	(media literacy AND health) AND (scale OR questionnaire OR instrument OR tool OR test)	220	6
	media literacy AND (scale OR tool OR questionnaire)	7	
	(media literacy AND health) AND (scale OR tool OR questionnaire)	20	
	(communications media) AND (information literacy) AND (health)	78	
WOS ^1^	(media literacy AND health) AND (scale OR questionnaire OR instrument OR tool OR test)	193	5
	(media literacy AND health literacy) AND (tool OR questionnaire)	42	
	(media literacy) AND ((health)AND ((tool) OR (questionnaire) OR (scale)))	63	
Dialnet	Alfabetización mediática AND (escala OR cuestionario OR instrumento)	91	0
	Alfabetización mediática AND (escala OR cuestionario OR instrumento) AND salud	5	
Scopus	media AND literacy AND health AND (scale OR instrument OR questionnaire)	167	1

^1^ WOS = Web Of Science.

**Table 2:** Assessment of the level of bias in tool design according to the COSMIN Risk of Bias.

Author (Year)	PROM Design	PROM Relevance and Comprehensiveness	Pilot Test Desing	Comprehensibility of the Pilot test	Comprehensiveness of the Pilot Test	Final Assessment (the lowest)
Primarck et al. (2006) [32]	A ^2^	A	D	D	-	Doubtful
Page, Huong, Chi and Tien (2011) [35]	A	A	D	-	-	Doubtful
Page, Piko, Balazs and Struk (2011) [36]	A	A	D	-	-	Doubtful
Levin Zamir et al. (2011) [12]	V ^1^	V	A	D	-	Doubtful
Chen et al., (2017) [33]	A	A	I ^4^	-	-	Inadequate
Demir et al. (2019) [37]	A	D	- ^5^	-	-	Doubtful
Kim et al. (2021) [38]	V	D	V	A	D	Doubtful
Long and Yoon (2022) [39]	V	A	V	D	D	Doubtful
Fleary (2022) [34]	D ^3^	D	A	D	D	Doubtful
Nazarnia et al. (2022) [41]	V	A	V	A	A	Adequate
Li et al. (2023) [40]	V	V	V	V	A	Adequate
Jormand et al. (2023) [42]	V	A	I	-	-	Inadequate

^1^ V = very good; ^2^ A = adequate; ^3^ D = doubtful; ^4^ I = inadequate; ^5^ - = no record.

**Table 4:** COSMIN (Consensus-based Standards for the Selection of Health Status Measurement Instruments) summary results of the criteria of measurement of psychometric properties evaluated.

Instrument	Article	Structural Validity	Internal Consistency	Reliability	Measurement Error	Hypothesis Testing	Cross-Cultural Validity	Criterion Validity	Responsiveness
SML ^1^	Primack et al. [32]	?	+	?	?	+	?	?	?
SML in Vietnamese Adolescents	Page et al. [35]	?	+	?	?	+	-	?	?
SML in Hungarian Adolescents	Page et al. [36].	?	+	?	?	+	-	?	?
MHL ^2^	Levin-Zamir, et al. [12]	?	+	+	?	?	?	+	?
SSB-ML ^3^	Chen et al. [33]	?	+	?	?	?	?	?	?
Turkish SSB-ML	Demir et al. [37]	+	+	+	?	?	+	?	?
K-SMLS	Kim et al. [38]	+	+	?	?	?	-	?	?
C-SSB-ML	Long and Yoon [39]	+	+	+	?	?	+	+	?
Adolescent MHL	Fleary [34]	?	+	?	?	+	?	+	?
MeHLit ^4^	Nazarnia et al. [41]	+	+	?	?	+	?	?	?
Chinese-MeHLit	Li et al. [40]	+	+	+	?	+	+	?	?
C-19ML ^5^	Jormand et al. [42]	+	+	?	?	?	?	?	?
Summary	Sufficient ^6^	50%	100%	33.3%	0%	50%	25%	25%	0%
	Insufficient ^7^	0%	0%	0%	0%	0%	25%	0%	0%
	Indeterminate ^8^	50%	0%	66.7%	100%	50%	50%	75%	100%

^1^ SML = Smoking Media Literacy scale; ^2^ MHL = Media Health Literacy scale; ^3^ SSB-ML = Sugar-Sweetened Beverages Media Literacy scale; ^4^ MeHLit = Media Health Literacy; ^5^ C-19ML; ^6^ sufficient = +; ^7^ insufficient = −; ^8^ indeterminate = ?.

## Data Availability

No new data were created.

## Supplementary Materials

The following supporting information can be downloaded at: https://www.mdpi.com/article/10.3390/nursrep14040206/s1, Table S1: PRISMA 2020 checklist.

## Public Involvement Statement

No public involvement in any aspect of this research.

## Guidelines and Standards Statement

This manuscript was drafted against the Preferred Reporting Items for Systematic reviews and Meta-Analyses (PRISMA) statement [21].

## Appendix A. Summary Table of the Studies Included in the Review

**Table:**

Author (Year)	Title	Country	Design Sample	Objective	Results	Conclusions	Evaluation of the Study Report
Primack et al. (2006) [32]	Development and Validation of a Smoking Media Literacy Scale for Adolescents	USA	-Cross-sectional study -1690 students aged 14 to 18 years at a large Pittsburgh, PA, public high school	To develop a media literacy scale for smokers (SML) and to evaluate the reliability and criterion validity of the scale.	-Cronbach’s alpha = 0.78 -SML showed significant associations with current smoking (*p* = 0.01), but not norms (*p* = 0.42).	Measurement of media literacy on smoking demonstrates excellent reliability and concurrent criterion validity. Given the independent link between media literacy and smoking, this could be a promising tool for future tobacco control interventions.	STROBE: 18/22
Chen, et al. (2017) [33]	Development and Evaluation of the Sugar-Sweetened Beverages Media Literacy (SSB-ML) scale and Its Relationship With SSB Consumption	USA ^1^	-Cross-sectional study -293 adults in rural southwestern Virginia	Create a SSB-specific media literacy scale. Describe the psychometric properties of the scale.	SSB-ML showed acceptable-to-strong levels of internal consistency scores (Cronbach’s alpha = 0.89).	SSB-ML describes media skills across an adult population and it is an appropriate tool to predict consumption patterns.	STROBE ^2^: 19/22
Fleary (2022) [34]	Development and validation of the Adolescent Media Health Literacy scales: Rasch Measurement Model Approach	New York, USA	Cross-sectional study; 355 adolescents included in the research	Develop and validate test-based scales of adolescents’ MHL.	-α del KR-20 = 0.91	The action/reaction dimension did not show good convergent and criterion validity; therefore, this scale should not be used until further research on its psychometric properties is conducted.	STROBE: 18/22
Page et al. (2011) [35]	Smoking Media Literacy in Vietnamese Adolescents	Vietnam	-Cross-sectional study -2000 students (grades 10–12) of two high schools	To evaluate social media literacy (SML) among Vietnamese adolescents and explore its correlation with smoking behavior and susceptibility to future smoking.	-Cronbach’s alpha = 0.87 -While SML was linked to reduced smoking overall, no association was found with susceptibility to future smoking.	The correlation between smoking media literacy (SML) and decreased smoking highlights the necessity for additional research on SML, in other adolescent populations.	STROBE: 19
Page, et al. (2011) [36]	Media literacy and cigarette smoking in Hungarian adolescents	Hungary	-Cross-sectional study -546 students in grades 8 and 12	To evaluate smoking media literacy among Hungarian youth and ascertain its relationship with both current smoking behavior and susceptibility to future smoking.	-Cronbach’s alpha = 0.75 -While smoking media literacy was linked to reduced current smoking rates similarly to American adolescents, it did not correlate with susceptibility to future smoking.	Hungarian adolescents demonstrated lower smoking media literacy than their American counterparts. While smoking media literacy was associated with decreased current smoking rates similar to American adolescents, it did not correlate with susceptibility to future smoking.	STROBE: 19
Levin-Zamir et al. (2011) [12]	Media Health Literacy (MHL): development and measurement of the concept among adolescents	Israel	-Cross-sectional study. -1316 Israeli adolescents from public schools	Developed new scale: Media Health Literacy (MHL)	This new measure (MHL) had an internal reability and consistency with a Cronbach’s alpha = 0.74.	This study confirmed the usefulness of this new scale for measuring media health literacy (MHL).	STROBE: 20/22
Demir et al. (2019) [37]	Psychometric properties of the Turkish version of the Sugar-Sweetened Beverages Media Literacy scale for university students	Turkey	Methodological descriptive correlational study. -884 university students	To translate and adapt the Sugar-Sweetened Beverages Media Literacy scale to the Turkish language.	-Cronbach’s alpha was 0.86.	The Turkish version was a suitable measurement tool for the Turkish sample.	STROBE: 20/22
Kim et al. (2021) [38]	Psychometric Properties of the Korean Version of the Smoking Media Literacy Scale for Adolescents.	Korea	-Cross-sectional study -215 total adolescents from five high schools in the capital city of Korea	To cross-culturally modify the Smoking Media Literacy Scale and evaluate the validity and reliability of the Korean version of the revised Smoking Media Literacy Scale for Adolescents (K-SMLS).	-Cronbach’s alpha = 0.79	This study confirmed that the K-SMLS is a valid and reliable instrument to assess SML among Korean adolescents.	STROBE 21/22
Long & Yoon (2022) [39]	Psychometric properties of the Chinese version of the sugar-sweetened beverages media literacy scale for undergraduate	China	Cross-sectional study. -1044 students from two universities in China	-Translate and adapt from English to Chinese the C-SSB-ML scale. -Describe the psychometric properties of the revised Chinese version of the SSB-ML (C-SSB-ML) and evaluate its validity and reliability.	-Cronbach’s alpha of C-SSB-ML scale was 0.92. -The three-factor model was adequate.	The C-SSB-ML is a valid and reliable instrument. It is also an appropriate tool to use in studies with young people because it is feasible and teachable.	STROBE: 20/22
Li et al. (2023) [40]	Psychometric evaluation of the Chinese version of the media Health Literacy Questionnaire: A validation study	China	Cross-sectional study. -514 adults	Translate the Media Health Literacy (MeHLit) questionnaire into Chinese and assess its psychometric properties.	-Cronbach’s alpha was 0.85. -Validation factor analysis, content validity, and reliability were appropriate.	The Chinese-MeHLit scale has adequate psychometric properties among the Chinese public, so it can be used to evaluate media health literacy.	STROBE: 20/22
Nazarnia et al. (2022) [41]	Development and psychometric properties of a tool to assess Media Health Literacy (MeHLit)	Irán	Cross-sectional study; 213 adults admitted to the research	Design a new psychometric instrument to assess Media Health Literacy: MeHLit.	-The MeHLit questionnaire was the first tool to assess media health literacy in adults. -MeHLit was a valid and reliable tool to measure media health literacy regarding individuals’ skills to assess health-related messages. -Cronbach’s alpha was 0.91.	-MeHlit was a validate and reliable questionnaire to assess media health literacy.	STROBE: 20/22
Jormand et al. (2023) [42]	Developing and validation of COVID-19 media literacy scale among students during the COVID-19 pandemic	Iran	Cross-sectional study. -530 students from a medical university	Assess C-19ML’s psychometric features	Cronbach’s alpha was 0.86. For content validity, construct validity, reliability, and external validity, the results obtained were optimal.	C-19ML is a reliable and valid tool for measuring the level of COVID-19 media literacy.	STROBE: 20/22

^1^ USA = United States of America; ^2^ STROBE = Strengthening the reporting of observational studies in epidemiology.
